# Calcined magnesite as an adsorbent for cationic and anionic dyes: characterization, adsorption parameters, isotherms and kinetics study

**DOI:** 10.1016/j.heliyon.2018.e00838

**Published:** 2018-10-03

**Authors:** T. Ngulube, J.R. Gumbo, V. Masindi, A. Maity

**Affiliations:** a Department of Ecology and Resources Management, School of Environmental Sciences, University of Venda, Private bag X5050, Thohoyandou, 0950, Limpopo, South Africa; b Department of Hydrology and Water Resources, School of Environmental Sciences, University of Venda, Private bag X5050, Thohoyandou, 0950, Limpopo, South Africa; c Council for Scientific and Industrial Research (CSIR), Built Environment, Hydraulic Infrastructure Engineering, P.O BOX 395, Pretoria, 0001, South Africa; d Department of Environmental Sciences, School of Agriculture and Environmental Sciences, University of South Africa (UNISA), P. O. Box 392, Florida, 1710, South Africa; e Department of Applied Chemistry, University of Johannesburg, Johannesburg, South Africa; f DST/CSIR National Centre for Nanostructured Materials, Council for Scientific and Industrial Research (CSIR), Pretoria, South Africa

**Keywords:** Environmental science, Theoretical chemistry, Physical chemistry, Materials chemistry, Analytical chemistry

## Abstract

The ability of calcined magnesite for Methylene Blue (MB), Direct Red 81 (DR81), Methyl Orange (MO) and Crystal Violet (CV) dye removal was evaluated in this study. The experiments were designed to test the hypothesis that alkaline earth carbonates can remove dyes from water through a combination of sorption and coagulative reactions involving Mg^2+^. To achieve that, several operational factors like residence time, dosage, adsorbent concentration and temperature were appraised. The batch study proved that calcined magnesite is effective in the treatment of MB, DR81, CV and MO contaminated water and moreover it performed well in terms of color removal. The adsorption equilibrium data were analysed by the Langmuir, Freundlich, Dubinin–Radushkevich and Temkin isotherm models, and the Dubinin–Radushkevich and Temkin models were found to be the most appropriate fit to MB and MO dyes respectively. The adsorption kinetics process primarily followed the Elovich and Pseudo-second order model, a possible indication that chemisorption was the rate limiting step during the dye uptake process. With the adsorption–desorption cycle repeated four times, the calcined magnesite regeneration efficiency for DR81 and MO loaded dyes remained very high. According to the results of this study, it can be concluded that calcined magnesite can be used effectively for the adsorption of MB, DR81, CV and MO from wastewater.

## 1 Introduction

Wastewater from numerous industries contain synthetic dyes from paper, textiles, leather and plastics (Ngulube et al., 2017). Wastewater that is discharged into natural canals and watercourses from dye manufacturing industries is a serious environmental threat (Yang et al., 2018). Even small amounts of dyes are capable of coloring huge volumes of water, thereby influencing the aesthetic value and decreasing light penetration needed for aquatic plant photosynthesis. Moreover, a lot of dyes are poisonous or carcinogenic (Uyar et al., 2016; Santos and Boaventura, 2016; Zhou et al., 2014). Hence, the elimination of dyes from wastewater is of great importance for environmental safeguard. Methods have been developed for dye removal in wastewater streams such as precipitation (Gupta et al., 2013), oxidation (Ghoreishi and Haghighi, 2003), adsorption (Gupta and Suhas, 2009), coagulation (Yagub et al., 2014) and membrane separation (Gupta and Suhas, 2009). Nonetheless, adsorption is recognized to be one of the effective techniques to treat wastewater (Ngulube et al., 2017). Activated carbon has been promoted as a good adsorbent due to its great sorption capacity for the dyes (Bello and Ahmad, 2012; Ozer et al., 2012; Malarvizhi and Ho, 2010). However, the high cost of activated carbon has led scientists to seek for low-cost adsorbents.

The removal of dyes by magnesium compounds has been proven to be a good alternative to the common adsorbents used in the treatment of industrial effluents (Bouyakoub et al., 2011), especially since conventional adsorbents like activated carbon and alum are not effective at pHs found in common wastewaters (Gao et al., 2007; Wang et al., 2009). However, the mechanisms by which dyes are responsive to removal by this kind of treatment have not been widely studied (Gao et al., 2007; Tan et al., 2000; Lee et al., 2006). Particularly, the adsorptive-coagulating mechanism that was originally proposed by Leentvaar and Rebhun (1982), must be further established.

The aim of this study is then to offer some new understanding into the capability of calcined magnesite, to remove dyes from synthetic dye wastewater. The principal aim of this study was to determine its potential application in the removal of Direct Red 81 (DR81), Methylene Blue (MB), Methyl Orange (MO) and Crystal Violet (CV) from aqueous solution by employing physico-chemical processes of adsorption.

## 2 Experimental

### 2.1 Materials

Calcined magnesite used in this investigation was obtained from Folovhodwe Magnesite mine, South Africa. MB, MO and CV dyes were purchased from Rochelle chemicals, South Africa. DR81 was purchased from Sigma Aldrich, South Africa. MB, DR81, MO and CV have a maximum absorbency at wavelengths 664, 510, 505 and 590 nm respectively. Their chemical formulas are C_16_H_18_N_3_ClS.xH_2_O, C_29_H_19_N_2_O_8_S_2_, C14H14N3NaO3S and C25N3H30Cl respectively. HCl, KCl and NaOH were also supplied by Rochelle Chemicals, South Africa.

### 2.2 Preparation and characterization of calcined magnesite

Calcined magnesite was milled to powder (particle size <50 μm) for 15 min at 1000 rpm by a stainless-steel vibratory ball mill (Retsch RS 200, Germany). The powdered material was kept in a zip-lock plastic bag for further use. FTIR analysis of calcined magnesite before and after dye adsorption was done using a FTIR spectrometer (Bruker Alpha, Germany). The phase structure of calcined magnesite before and after dye adsorption was evaluated by a PANalytical X'Pert Pro powder diffractometer (PANalytical, Netherlands). The morphology of calcined magnesite was analyzed using a scanning electron microscope JEM – 2100 Electron Microscope (JEOL, USA). The solid addition method as explained by Izuagie et al. (2016) was used to carry out Point of Zero Charge (PZC). Thermal stability was analyzed by a thermo gravimetric analyzer (TGA Q500, TA instrument) under air atmosphere with a flow rate of 50 mL/min and a heating rate of 10 °C/min. The determination of surface area was done via Brunauer Emmett Teller (BET) analysis (Micromeritics Tristar II, Norcross, GA, USA).

### 2.3 Preparation of MB, DR81, MO and CV working solutions

The MB, DR81, CV and MO solutions were prepared by dissolving suitable amounts of dry dye powder accurately weighed on an electronic balance (RADWAG electronic balance, Wagi Elektroniczen, Poland) in deionized water (ELGA, Micra Veolia Water Solution and Technologies, United Kingdom) to prepare a 1000 mg/L stock solution. Solutions to be used during experiments were obtained by serial dilutions to get solutions at desired concentrations. The final concentration of MB, DR81, MO and CV were estimated for each sample absorbance at the wavelength corresponding to maximum absorption peak using a UV/VIS spectrophotometer (Thermoscientific Orion Aqua Matte 7000, China). A standard calibration curve, used for the transformation of absorbance information into concentrations for equilibrium studies, was plotted to calculate the dye concentration of the experiments.

The percentage removal was computed by Eq. (1):

(1) $$Percentage\;removal=\left(\frac{C_{o}-\;C_{e}}{C_{o}}\right)\times \;100$$

The capacity of adsorption, Qe (mg/g) was obtained using Eq. (2)

(2) $$Qe=\;\left(\frac{C_{o}-\;C_{e}}{m}\right)\times v$$

where Co and Ce are the initial and equilibrium dye concentrations in solution (mg/L), v is the solution volume (L) and m is the weight (g) of dry adsorbent used.

### 2.4 Batch adsorption experiments

#### 2.4.1 Adsorption of MB, DR81, MO and CV as a function of contact time

To evaluate the effect of contact time on MB, DR81, MO and CV the adsorption experiments were carried out by measuring 50 mL of 10 mg/L MB, DR81, MO and CV solutions into 250 mL glass Erlenmeyer flasks. Deionized water (ELGA, Micra Veolia Water Solution and Technologies, UK) was used to prepare all the synthetic dye solutions. A fixed quantity of adsorbent (1 g) was added to each flask and kept on a reciprocating table shaker (Labotec, Model 207, South Africa) then agitated for varying contact times (15, 30, 60, 90, 120, 180 min). After the equilibration time, the solutions were left to settle for 30 min and then the supernatant solution absorbance was recorded by a VIS spectrophotometer. The experiments were carried out in triplicate at room temperature (25 °C) and the mean values reported.

#### 2.4.2 Adsorption of MB, DR81, MO and CV as a function of dosage

To evaluate the effect of adsorbent dosage on MB, DR81, MO and CV removal, 50 mL of 10 mg/L dye solution was measured into 18, 250 mL glass Erlenmeyer flasks and 0.1 g, 0.2 g, 0.4 g, 0.8 g, 1 g and 2 g of calcined magnesite were added to each flask. The mixtures were then agitated at 250 rpm using a reciprocating shaker for 60 min. After the equilibration time, the solutions were left to settle for 30 min and then the supernatant solution absorbance was recorded by a VIS spectrophotometer.

#### 2.4.3 Adsorption of MB, DR81, MO and CV as a function of dye concentration

To evaluate the effect of initial MB, DR81, MO and CV concentration on the removal capacity of calcined magnesite, samples of 50 mL of 1, 10, 15, 20, 25 and 30 mg/L MB, DR81, MO and CV solutions were measured into 18, 250 mL glass Erlenmeyer flasks and optimum dosages of calcined magnesite was added to each flask separately. The mixtures were then agitated for 60 min at 250 rpm using a reciprocating shaker. After the equilibration time, the solutions were left to settle for 30 min and then the supernatant solution absorbance was recorded by a VIS spectrophotometer.

#### 2.4.4 Adsorption of MB, DR81, MO and CV as a function of temperature

To evaluate the effect of solution temperature on the removal capacity of calcined magnesite, samples of 50 mL of and optimum concentrations of MB, DR81, MO and CV solution were measured into 12, 250 mL glass Erlenmeyer flasks and optimum dosages of calcined magnesite were added to each flask separately. The mixtures were then agitated for 60 min at 250 rpm using the reciprocating water bath shaker set at specified temperatures (298, 308, 318, and 328 K) with each different 3 samples. After the equilibration time, the solutions were left to settle for 30 min and then the supernatant solution absorbance was recorded by a VIS spectrophotometer.

### 2.5 Adsorption isotherm studies

Analyzing equilibrium data assists in developing some mathematical models that are helpful in quantitatively describing results. When put together, the basic assumptions and equations of the equilibrium models give imperative data on the mechanisms of adsorption. The following models were used in this study.

#### 2.5.1 The Langmuir isotherm model

The Langmuir isotherm has been widely used to discuss various adsorbate–adsorbent combinations for both liquid and gas phase adsorptions (Langmuir, 1918). The linear Langmuir isotherm may be represented by Eq. (3):

(3) $$\frac{C_{e}}{q_{e}}=\left(\frac{1}{Q_{m}}\right)C_{e}+\frac{1}{Q_{m}b}$$

Where *Ce* is the equilibrium concentration (mg/L), *qe* is the amount adsorbed at equilibrium (mg/g), *b* represents the Langmuir isotherm constant (L/mg) and *Qm* is the maximum adsorption capacity (mg/g) for a complete monolayer coverage.

The separation factor ($R_{L}$) Hall et al. (1966) was calculated from Eq. (4):

(4) $${\text{R}}_{\text{L}\;}=\frac{1}{\left(1+bCo\right)}$$

The R_L_ value indicates the shape of the isotherm. R_L_ values between 0 and 1 indicate favorable adsorption, 0 indicates irreversible adsorption, 1 means linear adsorption while a value greater than 1 indicates an unfavorable adsorption.

#### 2.5.2 The Freundlich isotherm model

The Freundlich model is an isotherm commonly used to describe heterogeneous systems (Freundlich, 1906). The model is represented in linear form by Eq. (5):

(5) $$logQ_{e}=\;n\;log\;C_{e}+log\;K_{F}$$

*K*_*F*_ and 1/*n* are the Freundlich constants, describing the adsorption capacity and intensity respectively. The constants *n* and *K*_*F*_ are determined from the slope and the intercept of the plot $\;log\;Q_{e}$ versus $log\;C_{e}$. When the value of n lies between 1 and 10 it represents beneficial adsorption (Aljeboree et al., 2017).

#### 2.5.3 The Dubinin and Radushkevich isotherm model

Common isotherm Eqs. (6) and (7) used analyse the rectangularity degree of isotherms. Eq. (6) is the Dubinin Radushkevich as projected by Dubinin and Radushkevich (1947).

(6) $$lnq_{e}=-K_{DR}\varepsilon^{2}+ln\;q_{DR}$$

(7) $$\text{Where}\;\varepsilon =RT\;ln\left(1+\frac{1}{C_{e}}\right)$$

where $\;q_{DR}$ (mg/g) is the adsorption capacity, $K_{DR}\;\left({\text{mol}}^{2}/{\text{kJ}}^{2}\right)$ is a constant related to the sorption energy (mol/k/J), ε is the Polanyi potential, T is the absolute temperature in Kelvin, R is the universal gas constant (8.314 J/mol/K).

E, which is the mean free energy of adsorption for each molecule of the adsorbate is calculated by Eq. (8)

(8) $$E=\frac{1}{\sqrt{2K_{DR}}}$$

E (kJ/mol) is the mean adsorption energy indicative of the heat of adsorption, signifying a physical or a chemical adsorption process.

#### 2.5.4 The Temkin isotherm model

The Temkin model as represented by Eqs. (9) and (10) was also applied in fitting the experimental data. The Temkin isotherm adopts that the adsorption heat on the surface declines linearly with the coverage adsorbate - adsorbent interaction (Temkin and Pyzhev, 1940). The Temkin adsorption isotherm linear form is given by Eq. (9):

(9) $$q_{e}=\beta \;\ln\;\alpha +\beta \;\ln C_{e}$$

(10) $$\text{where}\;\beta =\frac{RT}{b}$$

T is the absolute temperature in Kelvin, R is the universal gas constant (8.314 J/mol/K), and *b* is the Temkin constant related to heat of adsorption (J/mol), β is Temkin constant related to maximum binding energy (J/mol) and α is the equilibrium binding constant (L/mg).

### 2.6 Kinetic studies

To determine the rate limiting step in adsorption processes, kinetics of adsorption in batch systems are studied (Han, 2006). To evaluate the adsorption kinetics of MB, DR81, MO and CV molecules, four adsorption kinetic models were used.

#### 2.6.1 The pseudo first-order equation

The pseudo first-order model defines adsorption in solid–liquid systems founded on solids adsorption capacity (Ho, 1995).

Linearly, the pseudo first order model (Eq. (11)) is given as:

(11) $$\log\left(q_{e}-q_{t}\right)=\text{log}\;\left(q_{e}\right)-\left(\frac{k_{1}t}{2.303}\right)$$

Where $q_{e}$ (mg/g) is the capacity of adsorption at equilibrium, $q_{t}$ (mg/g) is the capacity of adsorption at time *t*, and *k*_*1*_ (1/min) is the pseudo-first-order rate constant.

#### 2.6.2 The pseudo second order kinetics

The pseudo second-order rate equation has been used to study chemical adsorption kinetics on liquid solutions founded on solid phase adsorption (Tran et al., 2017). Linearly it is given by Eq. (12):

(12) $$\frac{t}{q_{t}}=\left(\frac{1}{q_{e}}\right)t+\left(\frac{1}{k_{2}q_{e}^{2}}\right)$$

where $k_{2}\;$ is the rate constant for pseudo second-order adsorption (g/mg/h) $k_{2}q_{e\;}$ or h (mg/g/h) is the initial adsorption rate.

#### 2.6.3 The Elovich model

The Elovich equation is satisfied in chemical adsorption processes and is suitable for systems with heterogeneous adsorbing surfaces (Wu et al., 2009). In reactions involving chemical adsorption of gases on a solid surface without desorption of the products, the rate decreases with time due to an increase in surface coverage (Aharoni and Tompkins, 1970). One of the most useful models for describing such activated chemical adsorption is the Elovich Eq. (13) (Juang and Chen, 1997) which is given by:

(13) $$q_{t}=\frac{1}{\beta }\ln\left(\alpha \beta \right)+\frac{1}{\beta }lnt$$

where *α* and *β* are constants. The constant α (mg/g/min) considered as the initial sorption rate and β (mg/g) is the desorption constant during any one experiment and qt (mg/g) is the amount of dye adsorbed at time t (min).

#### 2.6.4 The intraparticle diffusion model

According to Weber and Morris (1963), if the rate-limiting step is intraparticle diffusion, a plot of solute sorbed against the square root of the contact time should yield a straight line passing through the origin (Poots et al., 1976). The most-widely applied intraparticle diffusion Eq. (14) for adsorption systems is given by Weber and Morris (1963):

(14) $$qt=k_{id}t^{\frac{1}{2}}+C$$

Where *kid* is the intraparticle diffusion rate constant (mg/g/min) and *qt* is the amount of dye adsorbed at any time *t* (mg/g) and *C* is the intercept.

### 2.7 Regeneration studies

The regeneration potential of calcined magnesite was evaluated by using 0.01 M NaOH. An adsorption experiment was done using 2 g adsorbent in a 50 mL, 10 mg/L dye solution. The quantity of adsorbed dye was noted, and the adsorbent was dried in the oven for 12 h at 105 °C. Afterwards the adsorbent was soaked into 100 mL, 0.01 M NaOH solution and the mixture was centrifuged at 50 000 rpm for 15 min. The amount of dye desorbed into the solution was recorded. The adsorbent was again washed by 100 mL deionized water. The desorbed adsorbent was again dried in the oven for 12 h at 105 °C. The dried adsorbent was again used for another adsorption experiment as described above. The same procedure using the same adsorbent was repeated three times.

## 3 Results and discussion

### 3.1 Adsorbent characterization

#### 3.1.1 Point of zero charge (PZC) analysis

**Fig. 1:**
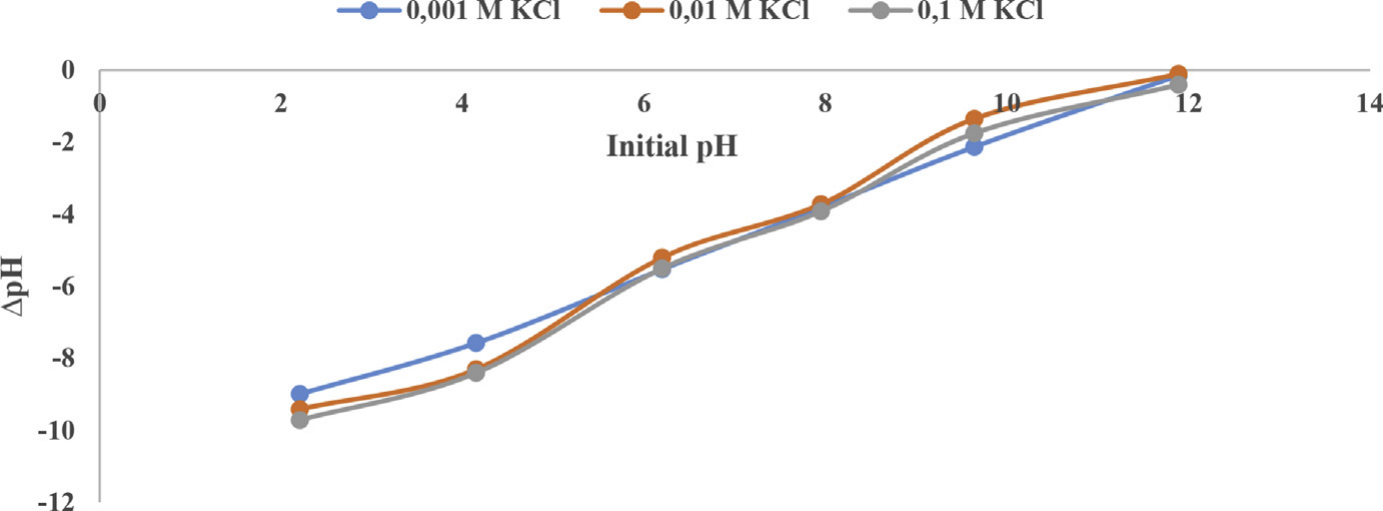
Point of Zero Charge of calcined magnesite.

The point of zero charge (PZC) designates a pH wherein the net total particle charge is zero. PZC is among the most significant parameters helpful in the description of variable-charge surfaces. When the adsorbent has a pH that is higher than its PZC, the adsorbent's surface will be negatively charged and consequently display the capability of exchanging cations, whereas, if its pH is below its PZC, the adsorbent will mostly retain anions (Ngulube et al., 2016). Fig. 1 shows that calcined magnesite has a PZC at around pH 12. Like observations were made by Han et al. (1998) on MgO. They pointed out that, the point of zero charge of magnesium oxide is well documented and is usually ± pH 12. Calcined magnesite is highly alkaline when in aqueous solution. During the adsorption experiments, after equilibration with the four different dyes, the final solution pH of the dyes ranged from around 10–11. pH results during the variation of contact time with the acidic and basic dyes are shown in Table 1:

**Table 1:** pH values before and after dye adsorption.

Dye	pH before adsorption	pH after adsorption
MB	10.63	11.99
DR81	8.24	11.86
MO	8.99	12.59
CV	10.39	11.70

The general observation was that despite being used in acidic or alkaline conditions, the introduction of calcined magnesite would change the aqueous solution to highly alkaline. Based on that, there was no further need to evaluate the influence of solution pH on the removal of the various dyes using calcined magnesite. Since this current adsorbent does not yield an acceptable pH for effluent discharge, it then becomes imperative for the wastewater to be neutralized by an acid to bring the pH of the contents into the acceptable range before disposal.

#### 3.1.2 X ray diffraction (XRD) analysis

**Fig. 2:**
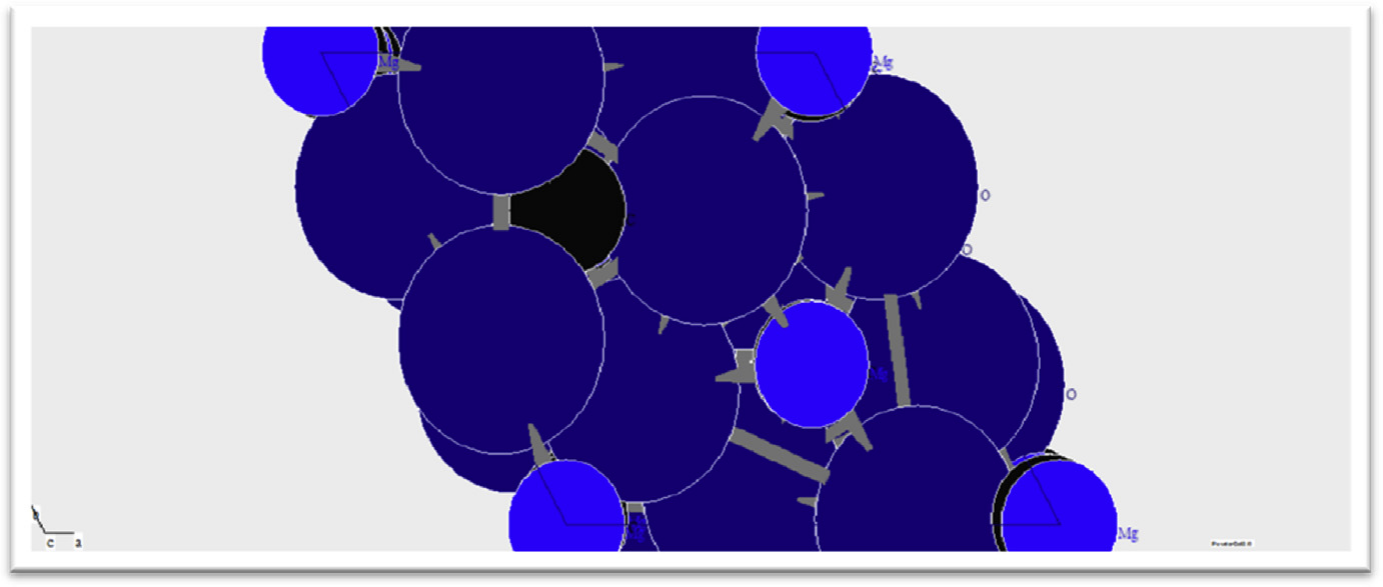
Crystal-chemical structure of calcined magnesite (Light blue represents Mg; Dark blue represents O; Black represents C).

The XRD patterns of the calcined magnesite sample showed that the major mineral constituent of the sample is periclase, which is basically a cubic form of magnesium oxide (MgO). Fig. 2 shows the crystal-chemical structure of calcined magnesite as analyzed by XRD.

**Fig. 3:**
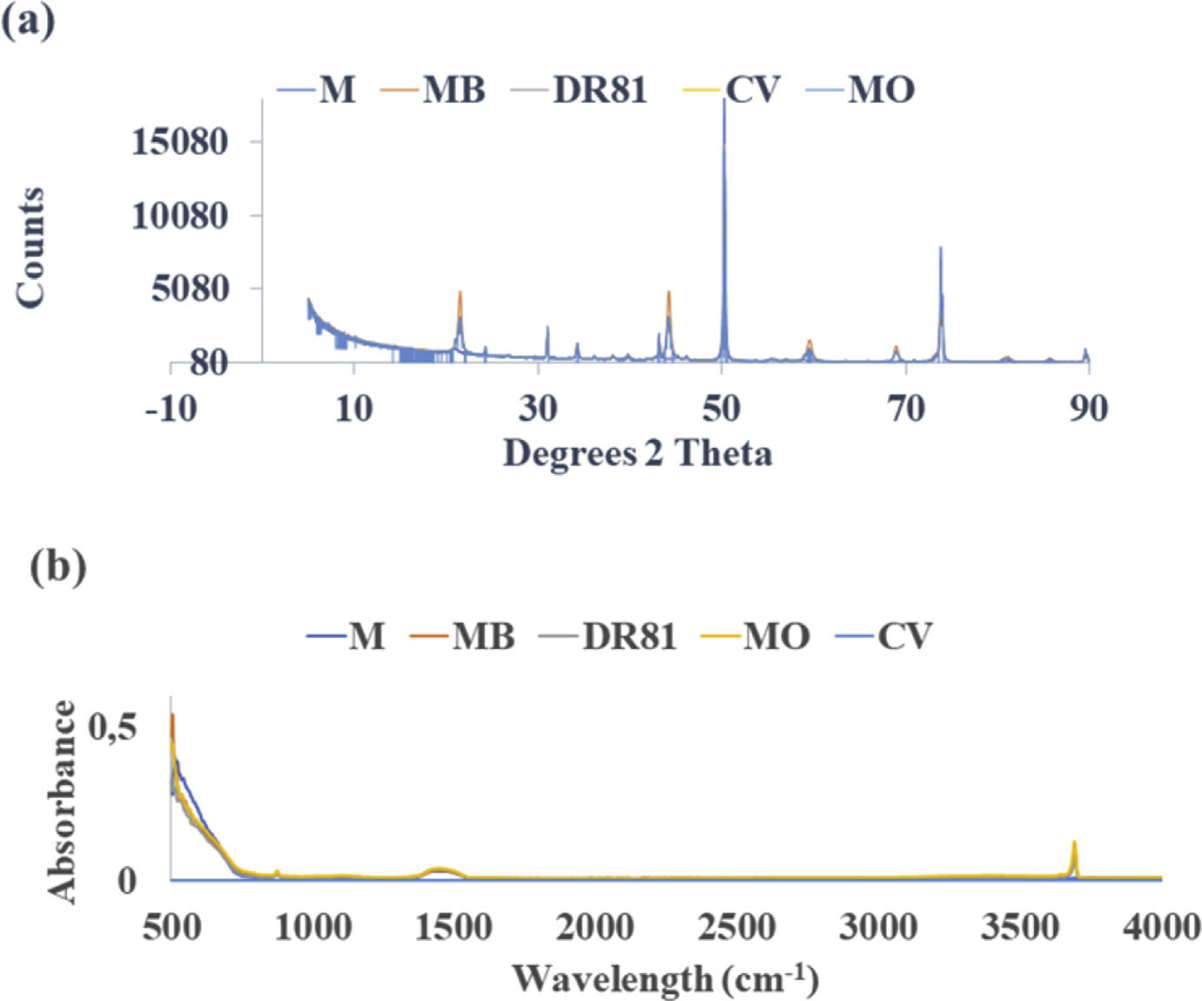
(a) XRD diffractogram of raw and MB, DR81, MO and CV reacted calcined magnesite (b) FTIR spectra of raw and MB, DR81, MO and CV reacted calcined magnesite.

XRD patterns of calcined magnesite are shown in Fig. 3(a). The sharp peaks in the XRD patterns imply good crystallinity of the sample. The identification of the patterns confirms the material to be largely periclase, this was also observed by Masindi and Gitari (2016) for cryptocrystalline magnesite. The diffraction patterns of the raw calcined magnesite and MB, DR81, MO and CV reacted calcined magnesite show that calcined magnesite had some minor but significant changes after MB, DR81, MO and CV adsorption. The raw calcined magnesite diffractogram shows the presence of periclase (MgO) in notable amounts at approximately (32, 45, 50 and 75) °2ϴ, quartz (SiO_2_) and periclase at about 32 °2ϴ and magnesite (MgCO_3_) at 38 °2ϴ. Initial studies on the crystal structures of various carbonate materials also reported essentially the same mineralogy as observed for the material used in this study (Effenberger et al., 1981). After MB, DR81, MO and CV adsorption, the intensity of the characteristic basal peak at 50.25 °2ϴ was reduced and slightly shifted to 51.24 °2ϴ. The shift and decrease in intensity indicate structural change in calcined magnesite is a result of the formation of brucite (Mg (OH)_2_). MgO can react with water (aqueous solution of dyes) to form Mg(OH)_2_ according to the equation

(15) $$MgO+\;H_{2}O\to Mg{\left(OH\right)}_{2}$$

Thus, the presence of brucite (Mg (OH)_2_) after adsorption of the dye could be a result of the hydroxylation reaction between the MgO with the aqueous solution of dyes. Brucite formation as a new material is also seen with new peaks at (21.57, 45.00, 60.66, 69.99 and 72.81) °2ϴ. This is evidence of a precipitation reaction taking place with hydroxides precipitating out of the solution. Similar results were obtained by Bouyakoub et al. (2011) when using MgCl to remove a reactive dye from textile waste water. Their results showed that brucite particles were formed when MgCl_2_ was applied to the textile wastewaters. This means that on contact with dyes, calcined magnesite reacted and changed in mineralogy as evidenced by the results. After reaction with DR81, there was also a notable increase of calcite from 1.79 to 7.42%. Another carbonate mineral in the form of dolomite CaMg(CO_3_)_2_ was also formed in small quantities on the DR81 and MO loaded calcined magnesite.

#### 3.1.3 Fourier transform infra-red analysis

The FTIR spectra for raw and MB, DR81, MO and CV treated calcined magnesite is shown in Fig. 3(b). Since the FTIR spectra of both the raw and MB, DR81, MO and CV treated calcined magnesite were more similar, only MB, DR81, MO and CV reacted calcined magnesite spectra were used to highlight the changes that occurred after adsorption. The FTIR spectroscopic studies confirm the results of XRD studies. After MB, DR81, MO and CV reaction, a brucite bending vibration corresponding to 3694 cm^−1^ was observed. The formation of brucite as seen on the FTIR spectrum corroborates the results reported on XRD, wherein new brucite peaks were seen. This new band could be used as evidence for adsorption of MB, DR81, MO and CV on calcined magnesite which further certifies the successful adsorption of MB, DR81, MO and CV by calcined magnesite. Similar developments of new bands were also observed on other studies after adsorbents interacted with the dye solution (Li et al., 2017; Wang et al., 2013). From the FTIR spectra of raw and MB, DR81, MO and CV reacted calcined magnesite it can be affirmed that these spectra are more similar, they only differ on the development of a new band at 3694 cm^−1^. Raw magnesite shows a system of bands characteristic of periclase stretching vibration corresponding to band 1450 and 880 cm^−1^ (Masindi et al., 2015). The vibration at 1450 cm^−1^, also supports the presence of carbonate materials including CaCO_3_ (calcite) and MgCO_3_ (magnesite). These results confirm the mineralogical composition as given by the XRD analysis which depicts the presence of periclase.

#### 3.1.4 Thermal gravimetric analysis (TGA)

**Fig. 4:**
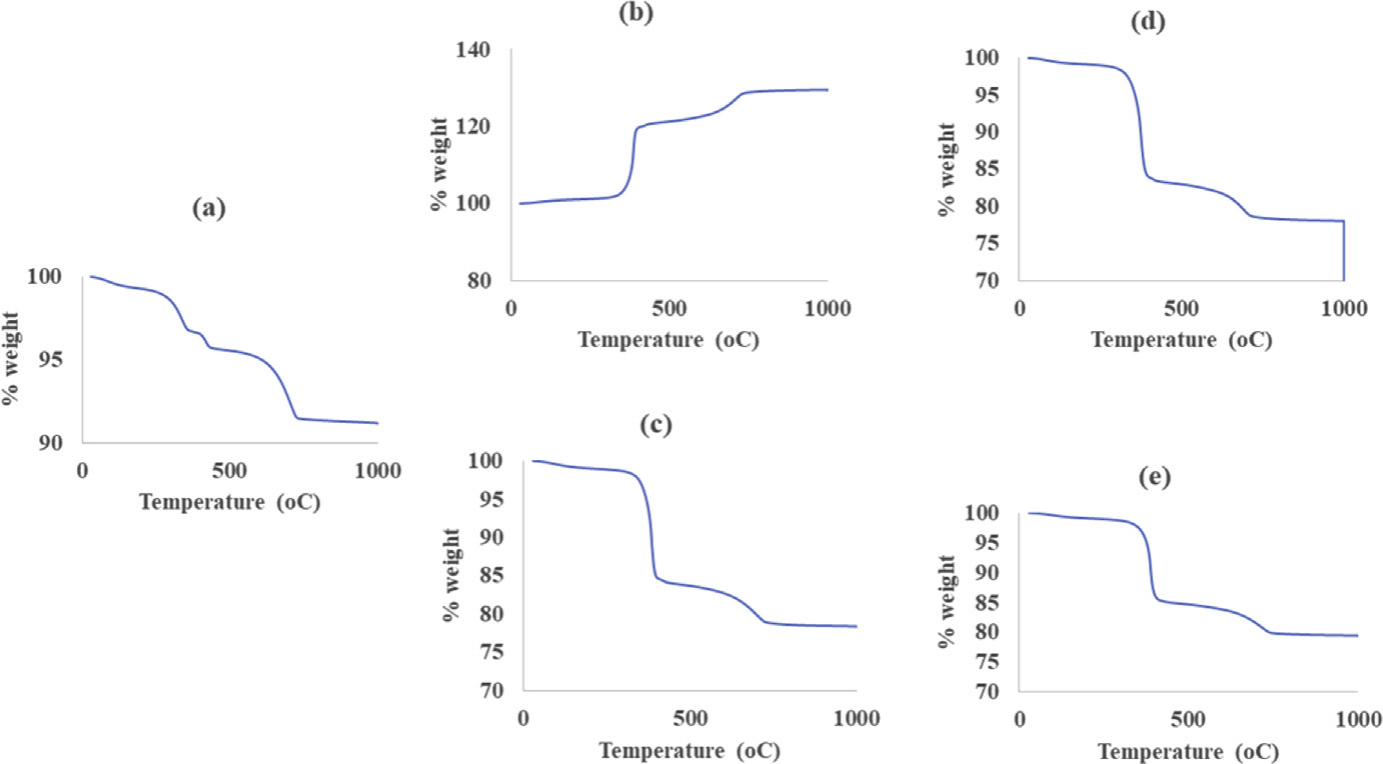
Thermograms of (a) – raw calcined magnesite and (b) – MB, (c) – DR81, (d) – MO, (e) – CV-adsorbed calcined magnesite.

The results of the thermal treatments of raw calcined magnesite and after adsorption of MB, DR81, MO and CV using the thermogravimetric analysis are shown in Fig. 4. The thermogram of raw calcined magnesite (a) shows a multi stage decomposition process. The decomposition process contains three phases with weight loss between 299 – 367 °C, 410–435 °C and 619–742 °C. The changes can be explained in terms of the decomposition of magnesite as per Eq. (16).

(16) $$Magnesite+dolomite+calcite+heat\to dolomite+calcite+MgO+CO_{2}$$

DR81 (c), MO (d) and CV (e) loaded thermograms showed a similar trend but different to that of the raw calcined magnesite (a) and MB (b) loaded calcined magnesite. This type of curve is exhibited by samples that undergo two mass losses, in this case, between 350 and 404 °C, and between 650 – 750 °C. On heating at temperatures above 350 °C, a rapid and continual weight loss to approximately 400 °C is observed in all 3 samples, after which, a steadier change is observed in the 650–750 °C temperature range. This weight loss up to 400 °C can be ascribed to hydroxylated water loss (Knowles et al., 2017) bound to the dye adsorbed calcined magnesite. The second decomposition phase can be due to possible transformation of the mineral phases via the loss of other volatile compounds.

There is an interesting observation with respect to MB loaded calcined magnesite (b). Contrary to (a), (c), (d) and (e), this thermogram shows weight gain rather than weight loss. This kind of curve is possible where there are numerous reactions as the temperature rises. The weight increase is a result of surface oxidation reactions (Loganathan et al., 2017), whereas the weight reduction with increase in temperature corresponds to material decomposition.

#### 3.1.5 Scanning electron microscopy (SEM) analysis

**Fig. 5:**
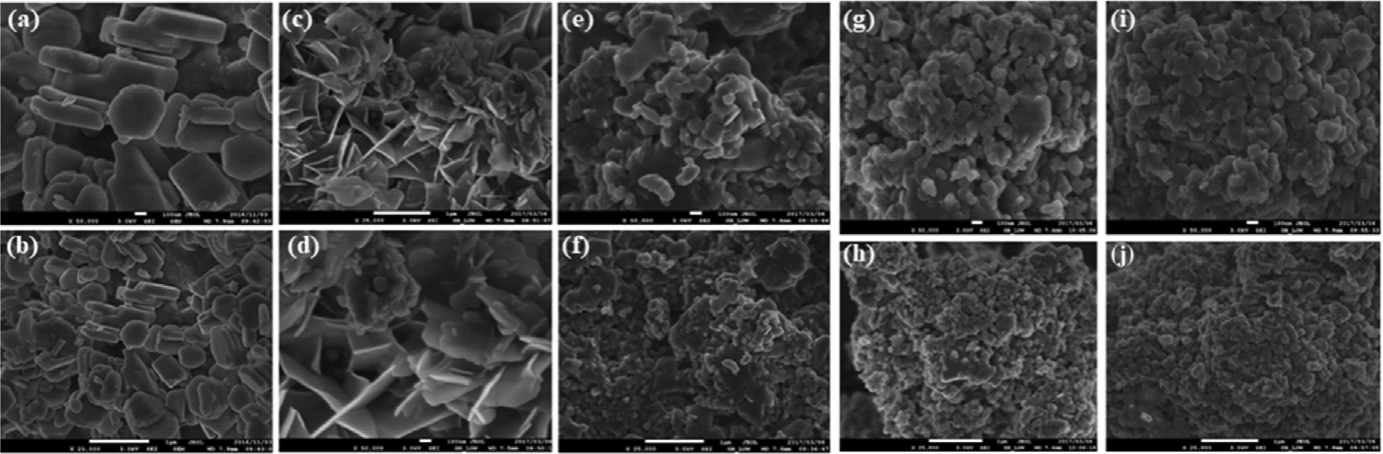
SEM images of (a & b) – raw, (c & d) – MB, (e & f) – DR81, (g & h) – CV and (i & j) – MO reacted calcined magnesite nanosheet particles at different magnifications (X 50 000 and 25 000).

The SEM images of raw and MB, DR81, MO and CV reacted calcined magnesite are shown in Fig. 5 at X 50 000 and X 25 000. The raw calcined magnesite images (a) and (b) appear to have definite hexagonal and rectangular shapes with different sizes. This is perfectly portrayed by image (a) which shows conspicuous quadrangular and rod like particles. On the contrary, images of the MB – (c) & (d), CV – (g) & (h) and MO – (i) & (j) reacted calcined magnesite have a significantly different appearance in both size and shape. After MB, CV and MO reaction the SEM images (c, d, g, h, i and j), particles appear to be broken down into small and medium size particles without a distinct shape. Worth noting is the eye-catching difference of DR81 reacted calcined magnesite images (e) and (f). Images (e) and (f) show irregular shaped flakes with sharp edges that are have characteristic leafy structure images. Most of the raw calcined magnesite particles are <200 nm in size. After the reaction with MB, DR81, MO and CV a reduction in particle sizes to <50 nm is observed, although some bigger sized particles were still seen. In the process of reacting in aqueous solution, MgO continuously dissolves and forms Mg (OH)_2_ (as evidenced by XRD results) on the surface of the parent particles. As the reaction proceeds, the larger particles of MgO are dissolved, and the particle size is gradually reduced. The surfaces of other particles seem to have been sufficiently transformed through aggregate particles created, yet others appeared to have a fibrous nature. This same observation was noted by Sarma et al. (2016). The transformation of the surface topography is evidence of MB, DR81, MO and CV being loaded onto calcined magnesite.

#### 3.1.6 Brunauer–Emmett–Teller (BET) analysis

Surface area is amongst the utmost significant physical properties that limit the quality and efficacy of an adsorbent. Variances of surface area and particle porosity seriously impact its performance. The surface area of particles ultimately determines its adsorption capacity. Table 2 shows that calcined magnesite has a BET surface area of 10.73 m^2^/g. After the adsorption of dyes, the surface area of calcined magnesite was reduced in the order: RDR81 > RMB > RMO > RCV. When compared to other clay-based adsorbents used for dye removal, calcined magnesite has comparatively lower surface area (Ngulube et al., 2017). The major reason for the high adsorption capacity of adsorbent materials is their high surface areas. If an adsorbent material has a higher surface area, it means that it will also have a higher adsorption capacity compared to clays with lower surface areas (Muller, 2010). However, this notion may not always be the case for some materials as exemplified by other studies (Chaari et al., 2015; Hai et al., 2015). The former study showed that halloysite had a surface area of 20 m^2^/g and therefore producing an adsorption capacity of 7.75 mg/g which is comparable to the present study. However, the latter study by Hai et al. (2015) shows that acid activated kaolinite had an exceptional high surface area of 358.6 m^2^/g but it produced a low adsorption capacity of 12.36 mg/g. Therefore, it can be established that other characteristics of an adsorbent material other than its surface area played a vital role in the adsorption of the four dyes.

**Table 2:** Surface areas of raw (R), MB, DR81, MO and CV reacted calcined magnesite nanosheet.

	Single point surface Area (m^2^/g)	BET surface area (m^2^/g)	Langmuir surface area (m^2^/g)
R	10.5895	10.7305	14.6564
RMB	8.1462	8.2205	11.1543
RDR81	9.4009	9.4891	12.8352
RCV	6.3461	6.3613	8.6613
RMO	6.4828	6.4434	8.7557

### 3.2 Optimization of adsorption conditions

#### 3.2.1 Contact time

**Fig. 6:**
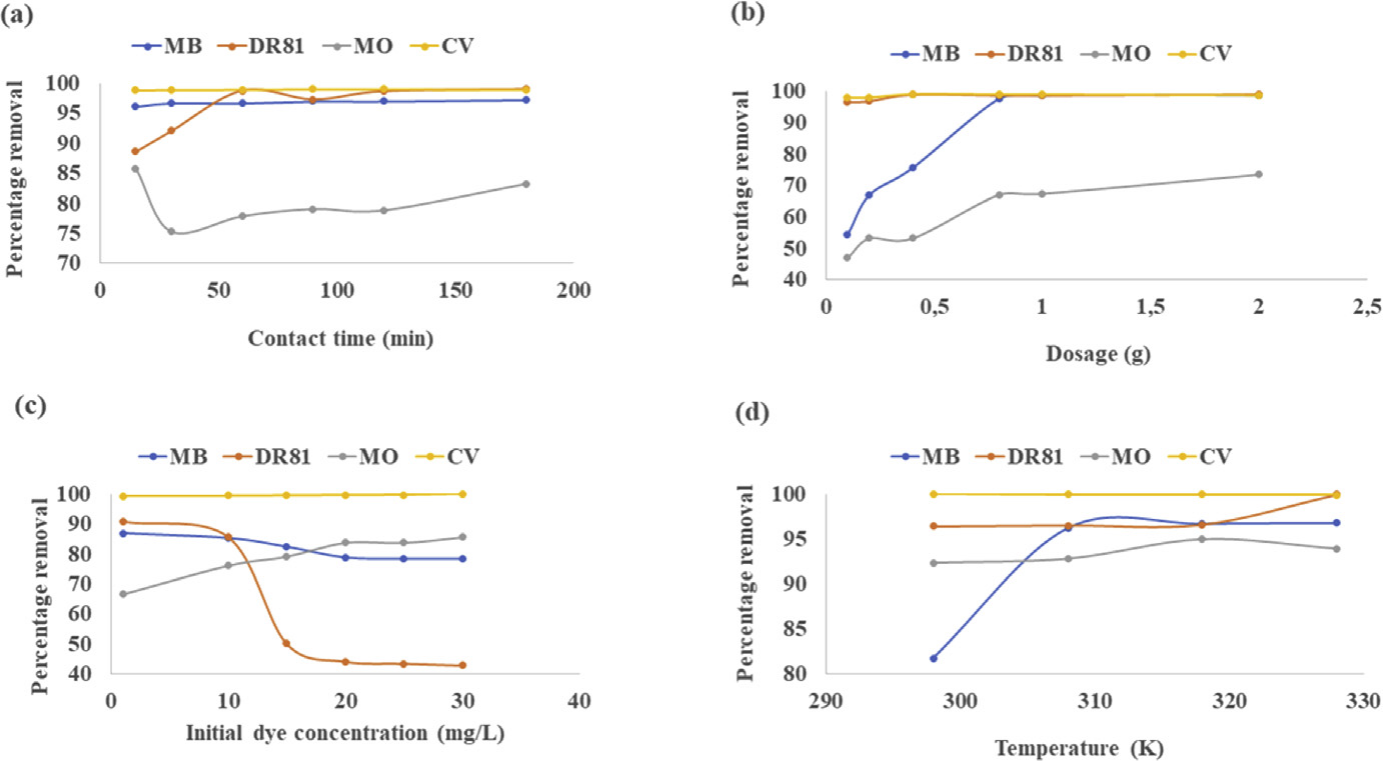
Removal of MB, DR81, MO and CV by calcined magnesite as a function of (a) – contact time; (b) – dosage; (c) – initial dye concentration (d) – temperature.

The rate of contaminants removal by adsorbents gives data that is essential in the prediction of the time needed to treat contaminated water consequently giving information on adsorption efficiency of the adsorption system (Yagub et al., 2014). Percentage dye removal with time is presented in Fig. 6(a). The graph shows that removal capacity of calcined magnesite increases slightly by increasing contact time for MB, DR81 and CV. For MO, there was a slight fall at 30 minutes and a rise again to the general trend after 60 minutes. The high percentage noted at first for MO dye could be due to all active sites taking up the dye ions at the initial stages. For all dyes, after 60 minutes of contact time, the percentage removal dropped, and no significant further change was observed till 180 min, hence 60 minutes of contact time was chosen as the optimum contact time for further experiments. The hypothetical justification to this trend can be credited to the amount of active adsorbent sites (Ismail and AbdelKareem, 2015). The rapid removal rate at the early stage is likely due to the accessibility of a great number of active sites on the surface of the adsorbent. Alike results were observed on other adsorbents reported for dye removal (Elmoubarki et al., 2015; Amuda et al., 2014).

#### 3.2.2 Adsorbent mass

Fig. 6(b) shows MB, DR81, MO and CV dye adsorption as a function of calcined magnesite dosage. Clearly, from Fig. 6(b), increasing the dosage of the adsorbent also increases the percentage dye removal. It is expected that increasing the dosage of the adsorbent increases the amount of available adsorption sites available to take up more dye molecules hence resulting in the percentage removal of dyes also increasing (Sarma et al., 2016; Chaari et al., 2015). Rehman et al. (2013) also reported on a direct association between percentage dye removal and dosage of the adsorbent. The percentage removal increased from 67 to 94, at a dosage of 0.3–1.5 g/L. A similar adsorption pattern with adsorbent amount was also given by Mane and Babu (2011) and Safa and Bhatti (2011). Adding the adsorbent quantity, increases the obtainable adsorption spaces to a definite level against a certain number of dye molecules (Ghaedi et al., 2011). The optimum dosages of 0.1 g for DR81 and CV and 2 g for MB and MO were chosen for the following experiments.

#### 3.2.3 Initial dye concentration

The trend in the percent MB, DR81, MO and CV removal with initial dye concentration is reported in Fig. 6(c). The percent CV and MO removal was directly proportional to the initial concentration within the evaluated initial concentration range of 1–30 mg/L. Hence CV and MO concentration was the sorption rate driving force at the given concentration range. A similar trend was reported by Rehman et al. (2013) on the removal of Brilliant Green dye by red clay. The authors revealed that the process of adsorption was directly proportional to the concentration of dyes as it drove the mass transfer rate below a greater gradient of concentration between the dye solution and the surface of clay. The same elucidation was reported by other authors including Aroguz et al. (2008) and Auta and Hameed (2012). On the contrary, MB and DR81 percentage removal declined with the increased initial dye concentration indicating that MB and DR81 adsorption relied on the number of actives sites that are available for taking up MB and DR81 ions. Similar trends of reduced adsorption capacity by increasing initial dye concentration were also described before (Tahir et al., 2016; Ullah et al., 2013).

#### 3.2.4 Temperature

The influence of temperature on MB, DR81, MO and CV removal by calcined magnesite is presented in Fig. 6(d). Experimental results show that as temperature was increased from 298-328 K, percentage dye removal slightly increased for MB and DR81 dyes. The improved dye removal with increase in temperature may be credited to kinetic effects due to enhanced diffusion of molecules or it can be attributed to new adsorption sites being “activated” (Zhou et al., 2014) on calcined magnesite at higher temperature. It seemed that the increase of temperature improved the diffusivity of dye molecules on water causing the increase of their movement into the pores of calcined magnesite (Ngulube et al., 2017). For MO and CV dye, it was observed that the differences in temperature had a negligible influence on the dye removal. Sivakumar et al. (2014a,b) also reported an insignificant percent dye removal at the different evaluated temperatures. Based on that, it was concluded that there was no need to adjust the temperatures for dye removal experiments hence 298 K (room temperature) was fixed as the optimum temperature to be used in subsequent experiment as this temperature is common for many water resources.

### 3.3 Isotherm studies

The batch adsorption studies were designed to evaluate the efficiency of calcined magnesite for the removal of MB, DR81, MO and CV from aqueous solution. The equilibrium data obtained was used to analyze the adsorption systems in view of the Langmuir, Freundlich, Dubinin Radushkevich and Temkin isotherm models. The isotherm constants of Eqs. (3), (4), (5), (6), (7), (8), (9), (10) are very valuable factors used to predict adsorption capacities and mass transfer relations to envisage the design and plan of batch reactors (Thilagavathy and Santhi, 2014). The equilibrium data for calculating isotherm constants were obtained by evaluating the effect of MB, DR81, MO and CV initial concentration on percentage dye removal. The isotherm model's validity was confirmed by comparing the R^2^ values and comparison of calculated and experimental adsorption capacities. Examination of the data shows that all the four adsorption models could not perfectly describe the uptake of the dyes. However, the Dubinin Radushkevich and Temkin isotherms fitted better to MB and MO adsorption data respectively compared to all other isotherm models tested though the R^2^ values of Freundlich and Dubinin Radushkevich models for MB adsorption were close to each other. Hence both isotherms may be held responsible for guiding MB adsorption, but it is also note worth noting that the best fit also means that both experimental adsorption capacity and calculated adsorption capacity are close to each other. In this case, the Dubinin Radushkevich isotherm adsorption capacity was closer to experimental adsorption capacity than the Freundlich hence the choice of the Dubinin Radushkevich as the best fit. The Dubinin Radushkevich isotherm is generally applied to express the adsorption mechanism with a Gaussian energy distribution onto a heterogeneous surface (Dubinin, 1960) whereas the Temkin isotherm assumes that the bonding energy of adsorption decreases linearly with increasing surface coverage (Temkin and Pyzhev, 1940). From the calculated isotherm model parameters for the Dubinin Radushkevich model, provided in Table 3 the mean free energy (E) was found to be 510.93 KJ/mol indicating a chemisorption process for MB onto a heterogenous surface. If there are any adsorbate – adsorbate interactions, they are best described by the Temkin isotherm. The Temkin isotherm plots for MO adsorption on calcined magnesite was linear with an R^2^ of 0.94 demonstrating that adsorbate – adsorbate and adsorbate – adsorbent interactions both control the dye removal process. The parameters of the Temkin model are presented in Table 3 show a higher value for b (5402.47 J/mol) which is an indication of the heat of sorption signifying a chemical adsorption for MO uptake.

**Table 3:** Parameters of the adsorption isotherms for the system of calcined magnesite.

	MB	DR81	MO	CV
Langmuir	R^2^ = 0.003	R^2^ = 0.4644	R^2^ = 0.3888	R^2^ = 0.0028
Freundlich	R^2^ = 0.9359 Qm exp = 0.39 (mg/g) KF = 0.0947 (mg/g) n = 1.0189	R^2^ = 0.0348	R^2^ = 0.8079	R^2^ = 0.0627
Dubinin Radushkevich	R^2^ = 0.9559 Qm exp = 0.39 (mg/g) Qm cal = 0.271 (mg/g) KDR = 0.0000019 (mol^2^/Kj^2^) E = 510.93 (kJ/mol)	R^2^ = 0.0304	R^2^ = 0.8588	R^2^ = 0.0627
Temkin	R^2^ = 0.8975	R^2^ = 0.0778	R^2^ = 0.9399 β = 0.4586 (J/mol) α = 0.7523 (L/mg) b = 5402.47 (J/mol)	R^2^ = 0.0627

### 3.4 Kinetic studies

To study the adsorption mechanisms like chemical reaction and mass transfer, four kinetic models were used to analyze the rate data. The adsorption parameters of the four kinetic models are presented in Table 4.

**Table 4:** The pseudo first order, pseudo second order, intra particle diffusion and Elovich kinetic model parameters for adsorption of MB, DR81, MO and CV onto calcined magnesite.

Parameter	MB	DR81	M0	CV
**Pseudo first order**				
R^2^	0.7930	0.0893	#N/A	#N/A
**Pseudo second order**				
Qe (mg/g) experimental	0.3932	12.5561	0.6416	14.9907
Qe (mg/g) calculated	0.2271	14.8148	0.4291	0.5209
k_2_ (g/mg/h)	0.0069	−1.10E^+17^	0.0001	0.0271
R^2^	0.9600	1	1	0.9898
**Intraparticle diffusion**				
Kid (mg/g/min)	0.2457	0.2306	0.7871	1.0056
C	0.8406	1.0834	0.3175	0.2486
R^2^	1	1	0.9999	1
**Elovich**				
α (mg/g/min)	1.3692	1.9050	0.5186	0.2854
β (mg/g)	1.713	1.8308	0.5239	0.4171
R^2^	1	0.9999	0.996	1

The batch adsorption system examined in this study aims to determine if it is suitable to be applied in treating field wastewater. The performance and cost of adsorbents including the mode of application are significant factors controlling the process efficiency (Zhou et al., 2015). For that reason, the adsorption capacity and the time taken to reach equilibrium become the most imperative parameters to determine in such studies. The adsorption rate was tested using four kinetic models to understand possible mechanisms involved in the adsorption process. The conformity between experimental data and the model predicted values was expressed by the correlation coefficients R^2^ (R^2^ values close or equal to 1). A relatively high R^2^ value indicates that the model successfully describes the adsorption kinetics. The parameters of the four kinetic models are presented in Table 4. The pseudo first order model yielded relatively low R^2^ values hence its applicability was dismissed. However, the other three models gave high R^2^ values in the order; intra particle diffusion > Elovich > pseudo second order as shown in Table 4. For the intra particle diffusion model to be valid, the plot of Qt versus t^0.5^ should be linear and pass through the origin (Guo et al., 2015). The fitting results (provided in the supplementary material) shows a linear regression, however, the plot did not pass through the origin (C≠ 0) which contradicted the validity of the intra-particle diffusion model suggesting that the adsorption of the dyes was not entirely controlled by intraparticle diffusion (Ugbe and Ikudayisi, 2017).

The Elovich equation is satisfied in chemical adsorption processes and is suitable for systems with heterogeneous adsorbing surfaces (Aharoni and Tompkins, 1970). As shown by the R^2^ values indicated in Table 4, the Elovich equation was successfully used to describe second – order kinetics behavior that concurs with the nature of chemical adsorption, if the actual solid surfaces are energetically heterogeneous (Senthilkumar et al., 2010). The results from the Elovich model corroborates those from isotherm modelling because they confirm that adsorption took place on heterogenous surfaces as evidenced by the Dubinin Radushkevich and Freundlich model fit on MB adsorption. Moreover, the proven applicability of the Elovich model signified the role of chemisorption as probably one of the basic rates limiting mechanisms during the adsorption process. This also concurs with the best fit shown by the pseudo second order model. The pseudo second order model basically supports chemisorption (Ngulube et al., 2017). Consequently, it can be determined that the rate limiting step in the adsorption of the four dyes is possibly chemisorption involving valence forces, occurring possibly due to electron sharing and/or exchange between calcined magnesite and dye ions in solution. The calculated and experimental adsorption capacities for MB, DR81 and MO were also close to each other confirming the best fit of the pseudo-second-order model. However, there was a significant difference between the calculated and experimental adsorption capacity with regards to CV which could possibly mean that CV uptake was not an entirely by chemisorption as explained in the mechanisms of adsorption of different dyes on Section 3.6.

### 3.5 Regeneration studies

**Fig. 7:**
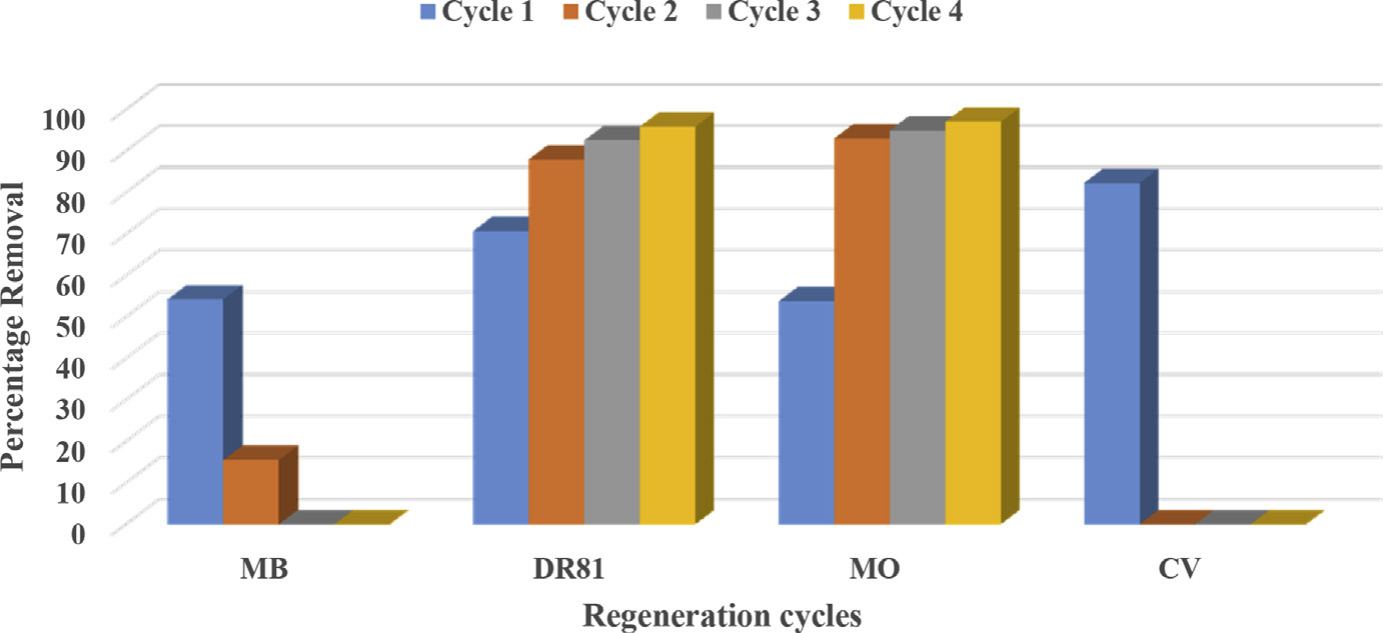
Regeneration test of calcined magnesite.

Excellent regeneration capability of an adsorbent is a crucial factor after the adsorption of dyes because it allows the reuse of the adsorbent. 0.1 M NaOH was used as an eluent in this study. The four dyes showed 2 different trends with each successive regeneration cycle. The general observation with regeneration experiments is that the more the adsorbent is regenerated and used for adsorption experiments, the more the adsorbent loses its adsorption capacity. This is the observation seen with MB and CV loaded calcined magnesite as seen in Fig. 7. Regeneration of CV loaded calcined magnesite proved ineffective just after one adsorption experiment whereas that of MB loaded calcined magnesite decreased to zero after 2 cycles. The decreased leaching of color from the CV loaded calcined magnesite is, on the other hand, a good characteristic, bearing in mind what would happen after disposal in landfills or reusing for other uses including integration in polymeric composites and construction materials (Ngulube et al., 2017). On the other hand, regenerating DR81 and MO loaded calcined magnesite with 0.1 M NaOH enhanced the adsorbent dye removal capacity with each successive cycle. The regenerated adsorbents were shown to be effective even after 3 cycles. It is shown that the percentage removal for each cycle was remarkably high proving that NaOH is an exceptional eluent to regenerate relevant adsorbents. Zhou et al. (2016) also recorded surprisingly high values of percentage removal with successive regeneration cycles. This behavior indicated that adsorption of DR81 and MO was chemical in nature. Adsorption was reversible and adsorbed DR81 and MO could be completely recovered with the alkaline solution. A possible explanation to this is that DR81 and MO dyes are acidic and reacting with alkaline solution leads to electrostatic interactions between the negatively and positively charged ions hence the reversible reactions.

### 3.6 Proposed mechanisms explaining the uptake of MB, DR81, MO and CV by calcined magnesite

Various factors like the adsorbent nature (physicochemical characteristics of the adsorbent) affect contaminants removal from water. The traditional theory of physical chemistry postulates that adsorption is a surface effect, Moreover, adsorption can be separated into chemical and physical depending on the nature of the interaction between the adsorbate and adsorbent (Saad et al., 2015). For physical adsorption, the main interactions are the Van der Waals' (VDW) forces between the adsorbent and adsorbate (Han et al., 2016). For chemisorption, the attraction force present between adsorbent and adsorbate have almost equal strength as chemical bonds and these are strong covalent bonds involving electron exchange. From the results obtained from the kinetic models, it is evident that MB, DR81, MO and CV were taken up by calcined magnesite particles via a chemical reaction. However, a further differentiation can be made between the acidic dyes (DR81 and MO) and basic dyes (MB and CV). The main mechanisms that controlled DR81 and MO uptake by magnesium nanoparticles include charge neutralization, electrostatic interaction and adsorptive coagulating mechanism. As shown in Fig. 3(a), the XRD study displays that the adsorbent is mainly made up of periclase which is principally magnesium hydroxide. It is also further revealed that the raw material is almost transformed into Mg(OH)_2_ precipitate in the color removal process. The precipitate structure offers a huge surface area for adsorption and a positive electrostatic surface charge, enabling the precipitate to be a significant and efficient coagulant. Hence, it can be inferred that calcined magnesite decolorizes the colored aqueous solution via adsorptive coagulating mechanism and charge neutralization.

When oxides and hydroxides of Mg and Ca ions are in solution they give out electrical charges which are involved in the dye removal processes. Charge neutralization is defined as a state in which the particles' net electrical charge in aqueous solution have been canceled by the adsorption of an equal number of opposite charges (Wang et al., 2009). Magnesium carbonate is among inorganic coagulants that usually work via charge neutralization. Once the metal-based coagulants are in aqueous solution, they dissociate, and metal ions are formed. In the case of magnesium carbonate, these liberated Mg ions and OH ions react with the dye molecules to make several polymeric and monomeric hydrolyzed species. Metal adsorption hydrolyzed products on the colloid surface causes charge neutralization bringing about van der Walls forces (O'Melia and Weber, 1972). Magnesium, with its divalent cations, is effective for neutralizing the negative charge of DR81 and MO dyes. According to a process respectively schematized as follows:

(17) $$Mineral\;ion\left(Mg^{2+}\right)\;\text{Positive}\;\text{charge}\;↔\;Dye\;organic\;fraction\;\left(SO_{3}^{2-}\right)\text{Negative}\;\text{charge}$$

(18) $$Mg^{2+}+SO_{3}^{2-}↔MgSO_{4}$$

There was an electrostatic interaction strong electrostatic attraction existing between the positively charged magnesium ion and the negatively charged dye molecules as shown by Eqs. (17) and (18), which could enhance the uptake of acidic dyes.

The nature of functional groups (hydrophobic/hydrophilic) present on the surface of the adsorbent lead to various interaction like hydrogen bonding and electrostatic attractions. This factor could be the one that played a role in the uptake of the basic dyes. As discussed in the FTIR characterization results, the bands at 3600–3700 cm^−1^ which are associated with OH groups adsorbed water shifted after dye adsorption indicating that the hydroxyl groups might have played an important role in the adsorption process. These modifications indicate the definite electrostatic, hydrogen bonding and dipole and ion-induced dipole forces among the functional groups of dye molecules and hydroxyls, Furthermore, as MB and CV molecules have C=C double bonds and benzene rings with π electrons, it could have π-π interaction with calcined magnesite in aqueous solution.

### 3.7 Performance evaluation

The maximum adsorption capacities of adsorbent materials for MB, DR81, MO and CV dye removal are listed in Table 5. It was observed that the maximum adsorption capacity of calcined magnesite is significantly comparative with the others reported in Table 5. The significance of the present study is to obtain maximum adsorption capacity at ambient temperature without modifying the natural adsorption conditions and the adsorbent itself. Most adsorbents with significantly high adsorption capacities are chemically modified but natural and/or raw materials have lower adsorption capacities. Nonetheless, calcined magnesite managed to achieve color removal as shown in the images provided in supplementary material.

**Table 5:** Comparison of adsorption capacity of various adsorbents for MB, DR81, MO and CV.

Adsorbent	Adsorption capacity (mg/g)	Dye	Reference
Calcined magnesite	0.39	MB	Present study
Calcined magnesite	12.56	DR81	Present study
Calcined magnesite	0.64	MO	Present study
Calcined magnesite	14.99	CV	Present study
Zn-MOF	0.75	MB	Sun et al.(2013)
Brazil nut shells	7.81	MB	Modesto et al. (2010)
Raw clay beads	58.02	MB	Auta and Hameed (2012)
Swelling clays	65	MB	Li et al. (2011)
Graphene oxide	714	MB	Yang et al. (2011)
*Argemone mexicana*	2.4	DR81	Khamparia and Jaspal (2016)
Bamboo Sawdust	6.43	DR81	Khan et al. (2012)
Pumice	10.56	DR81	Hossein and Behzad (2012)
*Balsamodendron Caudatum* wood waste	5.0354	DR81	Sivakumar et al. (2014a,b)
MgNiAl–CO_3_	118.5	MO	Zaghouane-Boudiaf et al. (2012)
Activated carbon	21.42	MO	Chennouf-Abdellatif et al. (2017)
Chitosan/MgO composite	60	MO	Haldorai and Shim (2014)
Mineral-based porous granulated material	80	MO	Wang et al. (2015)
TLAC/Chitosan composite	2.375	CV	Kumari et al. (2017)
Walled carbon nanotubes	90.52	CV	Sabna et al. (2015)
Subbituminous coal	6.25	CV	Schoonen and Schoonen (2014)
SnFe_2_O_4_@activated carbon magnetic nanocomposite	158.73	CV	Rai et al. (2015)

## 4 Conclusion

Calcined magnesite was successfully employed to remove of MB, DR81, MO and CV from aqueous solution. Results of the adsorption showed that MB, DR81, MO and CV dye removal improved with an increase in contact time and adsorbent dosage. Of the two kinetic models applied to the equilibrium data, the pseudo-second-order kinetic model could predict the adsorption kinetics well. Furthermore, the use of calcined magnesite is economically practical particularly because it can be recovered simple and reused afterwards. The results of this paper also provide more insight on the mechanisms involved when magnesium solutions are applied for discoloration of textile effluents. The mechanism of removal can be ascribed to electrostatic interaction, charge neutralization and adsorptive coagulating mechanisms. The application of magnesite for wastewater treatment seems to signify a capable substitute of conventional adsorbents because the optimum dose and production of sludge are very low compared to those accomplished by several natural adsorbents. After contacting dyes with calcined magnesite a colorless aqueous solution was observed. Such standards conform with the environmental standards on wastewater disposal in surface waters. Since the magnesite material showed an excellent dye removal capacity on a batch study, it become imperative to investigate its performance in a continuous fixed bed column mode in order assess the potential use in real wastewater. The employed calcined magnesite adsorbent proved to be effective in removing the dyes from water and demonstrated that it can be a hopeful answer to dye wastewater treatment problem.

## Declarations

### Author contribution statement

T. Ngulube: Conceived and designed the experiments; Performed the experiments; Analyzed and interpreted the data; Wrote the paper.

J.R Gumbo, V Masindi: Conceived and designed the experiments; Analyzed and interpreted the data; Contributed reagents, materials, analysis tools or data.

A. Maity: Conceived and designed the experiments; Analyzed and interpreted the data.

### Funding statement

This work was supported by the Director of Research and Innovation, 10.13039/501100008976University of Venda, 10.13039/501100001321National Research Foundation (NRF), 10.13039/501100004424Water Research Commission (WRC).

### Competing interest statement

The authors declare no conflict of interest.

### Additional information

No additional information is available for this paper.

## Appendix A Supplementary data

Revised Supplementary material Revised Supplementary material
