# Supplementary material for: Calcined magnesite as an adsorbent for cationic and anionic dyes: characterization, adsorption parameters, isotherms and kinetics study
Source: Heliyon. 2018 Oct 3;4(10):e00838. doi: 10.1016/j.heliyon.2018.e00838 (PMC6171090; doi:10.1016/j.heliyon.2018.e00838)
Supplement: Revised Supplementary material [file mmc1.docx]

**Supplementary material**

The intra particle diffusion model plot of Qt versus t^0.5^


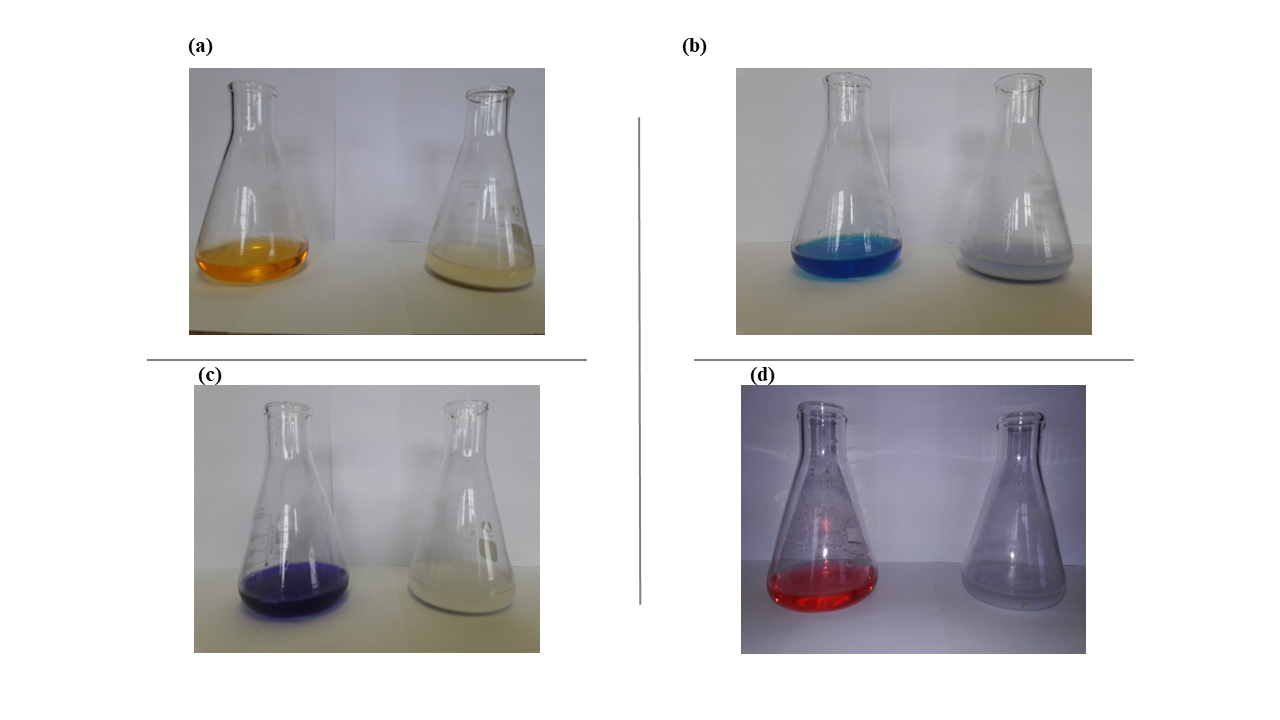


Images showing colour removal before and after adsorption by calcined magnesite on (a) – methyl orange; (b) – methylene blue; (c) – crystal violet; (d) – direct red 81
